# Development of a Credible Virtual Clinician Promoting Colorectal Cancer Screening via Telehealth Apps for and by Black Men: Qualitative Study

**DOI:** 10.2196/28709

**Published:** 2021-12-24

**Authors:** Danyell Wilson-Howard, Melissa J Vilaro, Jordan M Neil, Eric J Cooks, Lauren N Griffin, Taylor T Ashley, Fatemeh Tavassoli, Mohan S Zalake, Benjamin C Lok, Folakemi T Odedina, Francois Modave, Peter J Carek, Thomas J George, Janice L Krieger

**Affiliations:** 1 Department of Natural Sciences Bethune Cookman University Daytona, FL United States; 2 STEM Translational Communication Center College of Journalism University of Florida Gainesville, FL United States; 3 Harvard Medical School Harvard University Boston, MA United States; 4 Mongan Institute’s Health Policy Research Center Massachusetts General Hospital Boston, MA United States; 5 UF Health Cancer Center University of Florida Gainesville, FL United States; 6 Computer and Information Science and Engineering University of Florida Gainesville, FL United States; 7 Center for Health Equity & Community Engagement Research Mayo Clinic Jacksonville, FL United States; 8 Department of Health Outcomes and Biomedical Informatics College of Medicine University of Florida Gainesville, FL United States; 9 Family Medicine Medical University of South Carolina Charleston, SC United States; 10 Gastrointestinal (GI) Oncology Center University of Florida Gainesville, FL United States; 11 UF Health Medical Oncology – Davis Cancer University of Florida Gainesville, FL United States

**Keywords:** telehealth, digital health, eHealth, colorectal cancer, Black men, virtual human, technology, cancer screening, app, cancer, prevention, development

## Abstract

**Background:**

Traditionally, promotion of colorectal cancer (CRC) screening among Black men was delivered by community health workers, patient navigators, and decision aids (printed text or video media) at clinics and in the community setting. A novel approach to increase CRC screening of Black men includes developing and utilizing a patient-centered, tailored message delivered via virtual human technology in the privacy of one’s home.

**Objective:**

The objective of this study was to incorporate the perceptions of Black men in the development of a virtual clinician (VC) designed to deliver precision messages promoting the fecal immunochemical test (FIT) kit for CRC screening among Black men in a future clinical trial.

**Methods:**

Focus groups of Black men were recruited to understand their perceptions of a Black male VC. Specifically, these men identified source characteristics that would enhance the credibility of the VC. The modality, agency, interactivity, and navigability (MAIN) model, which examines how interface features affect the user’s psychology through four affordances (modality, agency, interactivity, and navigability), was used to assess the presumed credibility of the VC and likability of the app from the focus group transcripts. Each affordance triggers heuristic cues that stimulate a positive or a negative perception of trustworthiness, believability, and understandability, thereby increasing source credibility.

**Results:**

In total, 25 Black men were recruited from the community and contributed to the development of 3 iterations of a Black male VC over an 18-month time span. Feedback from the men enhanced the visual appearance of the VC, including its movement, clothing, facial expressions, and environmental surroundings. Heuristics, including social presence, novelty, and authority, were all recognized by the final version of the VC, and creditably was established. The VC was named Agent Leveraging Empathy for eXams (ALEX) and referred to as “brother-doctor,” and participants stated “wanting to interact with ALEX over their regular doctor.”

**Conclusions:**

Involving Black men in the development of a digital health care intervention is critical. This population is burdened by cancer health disparities, and incorporating their perceptions in telehealth interventions will create awareness of the need to develop targeted messages for Black men.

## Introduction

### Background

In the United States, colorectal cancer (CRC) is the second-leading cause of cancer-related death among both men and women. Despite improvements in incidence and mortality rates nationally, Black men bear a disproportionate burden of disease compared to the national average [1-3]. CRC incidence rates for non-Hispanic Black men in the United States are 58.3 per 100,000 compared to 46.9 per 100,000 among all men, with mortality rates of 25.9 per 100,000 compared to 17.7 per 100,000 among all men with CRC [2]. Multiple factors contribute to these inequities among Black men, including poorer access to preventive screening, aversion to colonoscopy, and limited knowledge of alternative screening modalities [4,5]. These inequities result in Black men reporting one of the lowest screening completion rates. To reduce CRC disparities among Black men, there remains a critical need to develop scalable CRC-screening promotion interventions that are tailored to meet the informational, cultural, and decision-making needs specific to Black men [6-10].

Home stool screening, through the fecal immunochemical test (FIT) kit, has been demonstrated as an effective alternative to colonoscopy [11-14]. FIT is a non-invasive stool test that requires no preparation, can be done in the comfort of the patient’s home, and is inexpensive. Previous patient outreach studies have shown that when given a choice to screen [2] for CRC, screening rates are greater for FIT compared to colonoscopy among Black patients (43% vs 26.6%) [12].

### Digital Health Interventions and Virtual Clinicians

Digital health interventions, including telehealth, are provisions of health and prevention resources to patients irrespective of their geographic location. The long-term benefits of telehealth indicate reductions in avoidable health care service utilization and related costs [14-17]. Short-term benefits include the ability to disseminate and personalize health information to patients in real time [14-16]. Telehealth interventions can effectively promote CRC screening but have been underutilized in the promotion of FIT screening. This study aimed to combine telehealth with FIT to overcome historical barriers associated with accessing CRC-screening resources among Black men.

The evolution of telehealth interventions now encompasses highly customized interactions with virtual clinicians (VCs). The VC featured in this study is entitled “Meet ALEX.” Agent Leveraging Empathy for eXams (ALEX) is an online virtual app using a visual representation of a health care provider that educates patients about the importance of CRC screening, identifies CRC-screening barriers, and provides a detailed description of the FIT kit. ALEX is interactive and replicates an in-person interaction using a combination of verbal and nonverbal behaviors. ALEX incorporates theory-driven conversational segments that include personalized risk information and barrier reduction strategies. Critically, ALEX can be tailored to reflect the demographic background (eg, race and gender) of the patient [18]. A growing body of literature has revealed demographic concordance between a health care provider and a racial/ethnic minority patient can increase adherence to recommended preventive testing and improve health outcomes [13,14]. Therefore, coupling a demographically concordant ALEX with FIT navigation offers promise to reduce CRC disparities among Black men. This method is only strengthened by incorporating Black men in the process of developing ALEX.

### MAIN Model Framework

A rigorous user-centered design (UCD) process was conducted to incorporate feedback from Black men to ensure ALEX was perceived as credible [19]. To provide a framework to interpret this data, we adopted the modality, agency, interactivity, and navigability (MAIN) model. The MAIN model offers an organizational framework to understand how a technology’s affordances (ie, its capabilities) affect a user’s perceptions of an interface [20]. The four broad categories of technological affordances are modality, agency, interactivity, and navigability. These affordances cue cognitive heuristics that lead to positive or negative credibility judgments after interacting with the technology [20-22].

In the MAIN model, each affordance is external to the content but affects the users’ perception of the media’s quality and credibility. The MAIN model has been applied to internet search engines, social media, video games, and virtual reality interfaces [23-25]. What is not always highlighted when the model is applied are the cues. Cues give way to some type of interaction between the designers and receivers in computer-mediated communications [26]. Cues are also known as effectors on users’ interactions and perceptions [20,24,26-28]. In this study, we apply the MAIN model to ALEX, an online app that can be assessed by any device that can connect to the internet.

### Objectives

The purpose of this study was to (1) conduct a three-phase UCD process with Black men to develop and iteratively improve ALEX and (2) utilize the MAIN model to better understand heuristic cues identified by the Black male participants that lead to positive credibility judgments of ALEX before clinical dissemination.

## Methods

### Study Design

Black men participated across a three-phase UCD process, which included (1) needs investigation, (2) prototype development, and (3) evaluation [29,30]. A detailed overview of the UCD process has been published previously, which incorporated transdisciplinary expertise across communication science, computer science, a community advisory board, and a clinical team to develop the ALEX app [19].

### Participant Recruitment

Black men from the ages of 50 to 73 years were recruited between January 2017 and November 2018 using a purposive sampling strategy [20]. To be eligible for the study, participants had to be active patients in the university health care system; this inclusion criterion was established, given that the ALEX character would be used in a subsequent clinical trial involving a similar population to promote use of FIT. Eligible participants were identified via university-affiliated community engagement organizations and local senior centers from rural counties of North Florida. Participants were recruited by study staff through phone, email, and in-person methods [19].

Focus groups were conducted in person across various locations throughout the community. Each moderated focus group lasted between 60 and 90 minutes. To ensure rapport with the participants, the moderator was also a Black male. Institutional review board approval was granted for this study, and the participants provided informed consent prior to being audio-recorded. At the beginning of each interview, participants were provided information about the overall aim of the study and how the study team would incorporate participant feedback to help develop the Black male VC in the ALEX app. Each participant received a $40 gift card as compensation for their time and effort.

### Data Collection

Feedback was collected across 8 focus groups to iteratively and systematically develop the Black male VC. Instead of recording the script and developing the interactive VC animation for the first set of focus groups, we obtained initial feedback on the still images of the VC and then applied a script and engaged in character development. The UCD ensured the involvement and perceptions of the Black men throughout the entire process, systematically tailoring the VC based on their feedback. Each focus group discussion began with identifying the participants’ familiarity, understanding, and knowledge of cancer, cancer screening, and virtual human technology. Participants in the first and second interviews viewed still images of the VC. Participants in the third, fourth, and fifth focus groups viewed iteration 1 of the VC prototype. Participants in the sixth and seventh focus groups viewed iteration 2 of the prototype, and participants in the eighth focus group provided feedback on the third and final iterations of the VC used in the ALEX app.

### Data Analysis

Audio recordings were transcribed verbatim and reviewed for accuracy. Transcripts were uploaded into NVivo (version 12.0; QSR International) to allow for electronic coding of the data. The data were analyzed using thematic analysis. Two coders (authors MV and DW) established an initial codebook with guidance from a senior author and expert in qualitative research (author JK). The codebook included a priori codes derived from the credibility literature and domains of inquiry from the interview guide. The full codebook is published in a previous publication [31]*.*

### Coding Procedures for the MAIN Model

**Figure 1:**
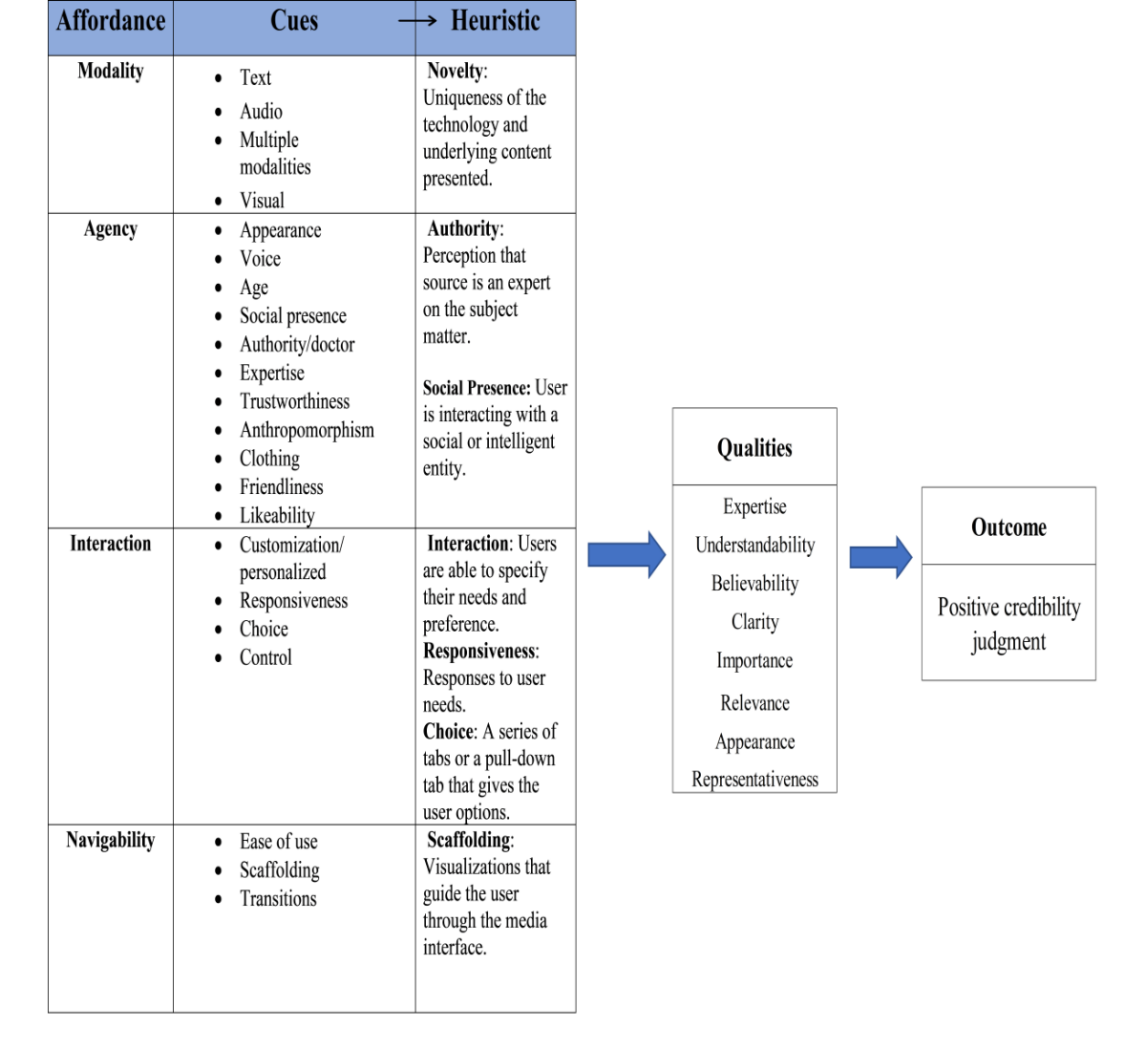
MAIN model affordance with the cues, heuristics, and qualities that lead to a positive credibility judgment for ALEX, the virtual health care assistant. MAIN: modality, agency, interactivity, and navigability; ALEX: Agent Leveraging Empathy for eXams.

Codes reflected theoretically informed components of credibility and guided a deductive coding approach for the presence of affordances, cues, and heuristics from the MAIN model, as seen in Figure 1. These cues prompted multiple heuristics such as social presence, authority, interaction, responsiveness, choice, control, and scaffolding, leading to an overall quality experience triggering enhanced credibility. The qualities associated with enhanced or positive credibility include uniqueness, trustworthiness, expertise, appearance, understandability, believability, clarity, importance, relevance, and representativeness. As shown in Figure 1, cues and heuristics were organized according to the affordance where modality examined how the content was conveyed through media (visual, text, audio, etc). Agency identified the source, that is, who or what was providing the content through the media interface (VC, text). Interactivity affordances identified cues that were associated with being in the presence of the digital media interface (the actions of clicking). Navigability examined the digital media interface’s ability to guide the user through the content (dialog boxes, links, etc).

During a line-by-line reading of the transcripts, emergent codes were added to the codebook through an inductive coding process. All codes were operationalized and assigned provisional definitions with exemplary quotes [31]. A formal coding framework was then established and used to determine interrater reliability (IRR). Two coders independently coded the transcript. An acceptable IRR was established by a κ coefficient above 0.8. The IRR was calculated using NVivo’s coding comparison query. Initially, 16 coding discrepancies were documented and sent to a third reviewer (JK) for feedback. After 3 rounds of coding, low κ scores (below 0.5) were determined to be due to unitization issues rather than disagreement on coding [27]. To address this, transcripts were segmented by both coders by isolating text in the transcript using box frames to create a unit. Each unit was coded in its entirety to ensure the coders would capture the same information from beginning to end. In the fourth and final round of coding, κ was above 0.8 for all codes, and the rest of the transcripts were coded.

## Results

### Major Findings

Qualitative focus groups (included 7 focus groups and 1 individual interview) were conducted with a total of 25 Black men. The 1 individual interview occurred because 2 of the invited participants rescheduled on the day of the focus group. Therefore, he was the only participant during his focus group [19,29,32]. Demographic information of the men recruited can be found in Table 1. In summary, Black men were between the ages of 50 and 73 years (median age 61 [6.12]), with income ranging from less than $10,000 to over $100,000 annually. Their education levels ranged from eighth grade level to postsecondary and professional degrees.

**Table 1:** Demographic information about the focus groups of Black male participants (N=25).

Demographics of focus groups of Black male participants (2017-2018)		n (%)
**Marital status**		
	Married	6 (24)
	Divorced	4 (16)
	Separated	6 (24)
	Single	4 (16)
	Widowed	1 (4)
	Did not answer	4 (16)
**Education level**		
	Grades 1-8	1 (4)
	Some high school	2 (8)
	High school graduate or General Educational Development (GED) certificate	8 (32)
	Technical, trade, or vocational school	0
	Some college or associate degree	5 (20)
	College graduate (BS, BA, or other 4-year degree)	3 (12)
	Postgraduate training or professional school	2 (8)
	Did not answer	4 (16)
**Employment status**		
	Full-time	2 (8)
	Part-time	5 (20)
	Retired	5 (20)
	Volunteer	1 (4)
	Unable to work due to disability	6 (24)
	Unemployed	3 (12)
	Did not answer	3 (12)
**Income level**		
	Less than $10,000	7 (28)
	$10,000-$19,000	5 (20)
	$20,000-$34,999	6 (24)
	$35,000-$49,999	1 (4)
	$50,000-$74,999	0
	75,000-$99,999	1 (4)
	$100,000 or more	1 (4)
	Preferred not to answer	2 (8)
	Did not answer	2 (8)

Four of the focus groups were exclusively Black men, with three focus groups including a combination of Black and White men (Table 2). Feedback from the Black men was extracted and analyzed from the combination groups, as also shown in Table 2. For the developmental iterations of ALEX, the results are presented according to the affordances associated with the MAIN model: modality, agency, interaction, and navigability.

**Table 2:** Summary of focus groups and the number of Black men who participated.

Black men only or combined Black and White men	Number of men (N=37), n (%)	Number of Black men (N=25), n (%)	Participant # of Black men	VC^a^ version
Black men only (group 1)	5 (14)	5 (20)	104-108	Still images
Black men only (interview)	1 (3)	1 (4)	109	Still images
Black men only (group 3)	4 (11)	4 (16)	14-17	Version 1
Black men only (group 4)	2 (5)	2 (8)	18, 19	Version 1
Combined (group 5)	4 (11)	1 (4)	73	Version 1
Combined (group 6)	6 (16)	4 (16)	119-122	Version 2
Combined (group 7)	7 (19)	1 (4)	126	Version 2
Combined (group 8)	8 (22)	7 (28)	147-152, 154	Version 3

**^a^**VC: virtual clinician.

### Iteration 1: Still Images of ALEX

#### Modality

**Figure 2:**
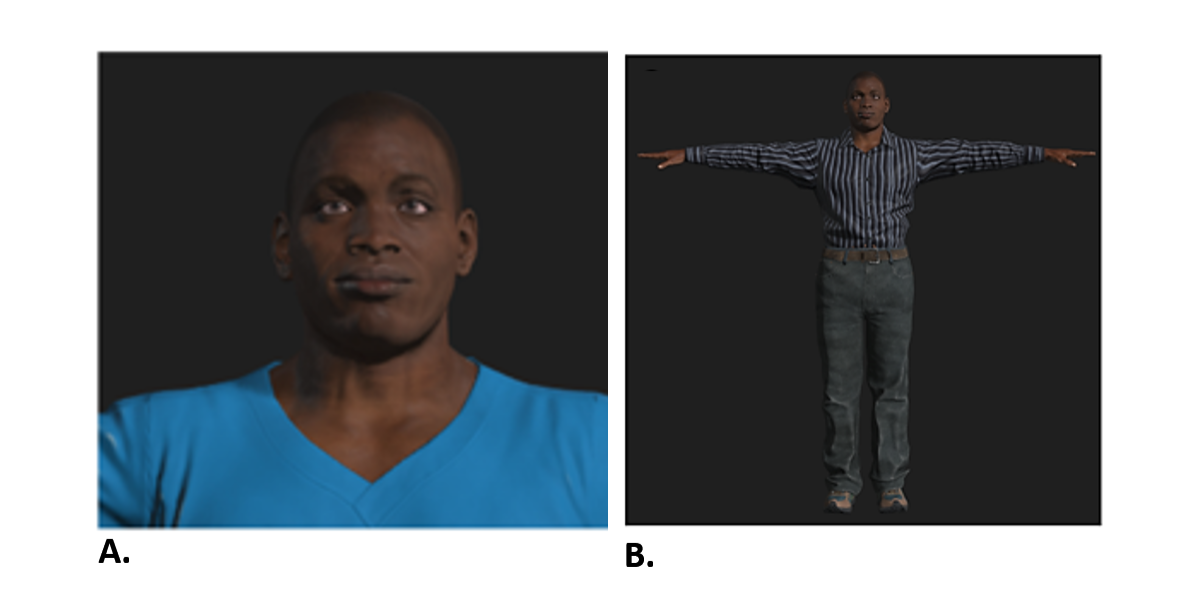
Near and far still images of the first iteration of the Black male ALEX character. A: the near iteration and B: the far iteration. ALEX: Agent Leveraging Empathy for eXams.

Two focus groups viewed near and far printed images of the Black male VC, as shown in Figure 2. While participants reviewed the still images, moderators played voice clips and explained the plan to deliver the VC through multiple modalities (audio, visual, and text). Participants expressed strong support for the multimodal approach to intervention development. For example, one participant stated,

So, for people who can't read, that would be perfect.

P107

#### Agency

Participants discussed agency in relation to how ALEX would function as a source of health information. Overall, participants expressed that obtaining health information from a VC was a new concept. These participants envisioned ALEX functioning like other trusted virtual apps, such as Google Maps or Siri, in which the VC would act like the Global Positioning System (GPS) for their CRC-screening journey. One participant noted,

I like that virtual human as long as they [are] just like maybe the GPS. They're gonna make sure you're right…And so that virtual human could be good if he's gonna make sure you stay on the path.

P105

For some, if they learned they were at high risk for CRC and needed to change modifiable behaviors to reduce their risk, as a result of information conveyed by ALEX, they would still seek a second opinion from their provider. One participant said,

I wouldn't accept it just because it was an app. You know, if the doctor told me that, if I knew my doctor and my nurse, and he told me that, you know, I'm feeling like he could probably sympathize, kind of urge me.

P105

#### Interaction

Predicting the interaction with the app gave the men a sense of pride, knowing that it could be a resource that would help facilitate a conversation with their doctors. One participant noted,

I could interact with it where I can be comfortable and go backward and forward and determining the severity of my situation, whereas to encourage me to go get medical assistance…do preventative maintenance.

P106

This cued the social presence–enhancing heuristic of the app, where the participants began to take ownership of it as a possible support system, as stated by one participant:

I’ll try it out. I mean, if me and that app—I'd be at the hospital real fast and the thing about that app, for me, familiarized me with some of details how to explain to the doctor what I think is the symptoms that I'm coming in contact with.

P104

Participants discussed other cues associated with the interaction with ALEX, including perceptions of the customization or personalization of the VC. One participant said,

It asks me for a certain thing, and I give it to them. Then it shows me my risk factors. Now, if it shows me something that I would determine to be—I would take—I…I—no questions about it. I'd go to the doctor to make doggone sure it is—you know, either to curtail it or to prevent it. It doesn’t matter.

P106

#### Navigability

The means of accessing ALEX and navigating through the interface was important and played a role in increasing trust and credibility. Knowing that ALEX was designed, developed, and would be delivered from their health care system was important, as stated by one participant:

All my information is through email. Now, I would have to make sure— like you were saying, you got to make sure where it's coming from. Now, I'll see something that's associated with Shand’s because that's where my doctors are at, and I click on it.

P108

### Iteration 2: First Video of ALEX

**Figure 3:**
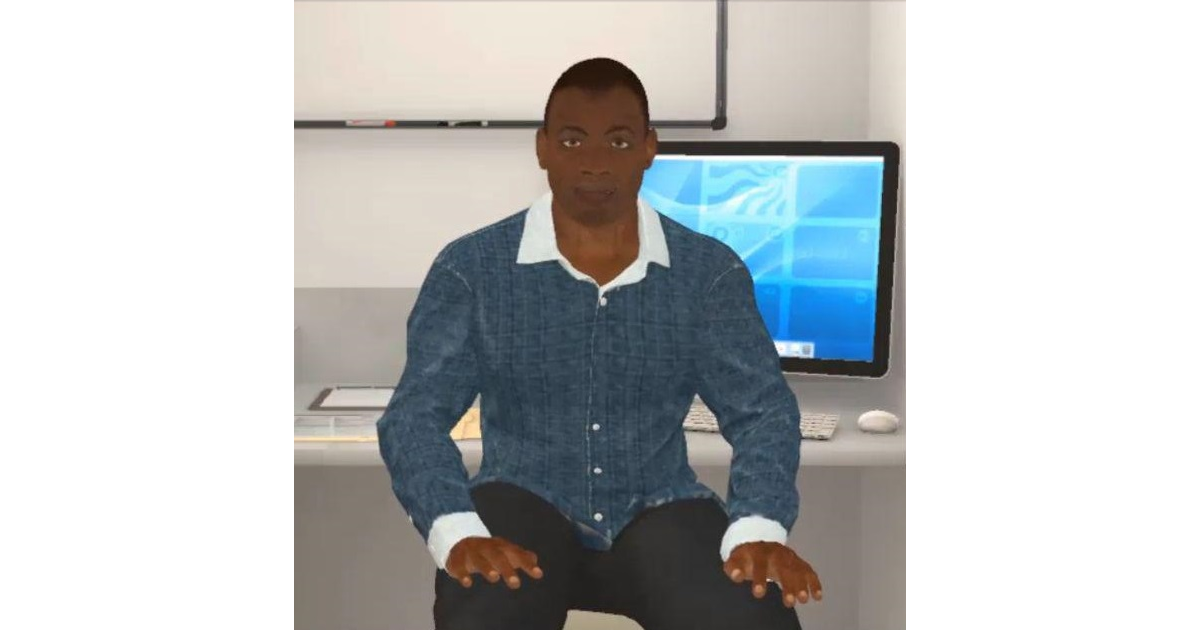
Image of the first video of the virtual health care assistant, ALEX. ALEX: Agent Leveraging Empathy for eXams.

In iteration 2, 3 focus groups engaged with ALEX on a Samsung smartphone. In this iteration, participants viewed a computer software program that created an initial rendering of the VC, as shown in Figure 3. The VC could speak to participants, and the app contained subtitles corresponding to the speech.

#### Modality

Overall, the multimodalities were useful to participants. One participant said,

I liked that it was really, uh, it gave you all the information, it was on a personal level. So, man, woman, no matter who you are, you can listen to and watch the app. And that it has also, the, the words on the bottom. So, if you don’t understand what the app is saying, you can also read it. That was really helpful.

P16

Credibility was essential to the success of the app so that patients would engage with it honestly. One participant stated,

We have to kind of win the confidence of the future patients that they [patients] will, though they are not in front of an actual doctor, they will tell the truth.

P18

#### Agency

The virtual character had low visual fidelity with casual clothing and minimal breathing animations. The background of the character was a 3D model of a health care provider’s office, as shown in Figure 3. Comments about agency reflected a range of verbal and nonverbal cues, including the character’s physical appearance. Specifically, ALEX’s appearance was not “human-like” enough, with one participant commenting,

It looked like a mummy…that threw me off…it was futuristic.

P73

Others described the VC as “cartoonish” and even “scary,” all of which diminished perceived credibility. In addition to comments about the physical appearance of the VC, other comments critiqued how the VC’s clothing should look like a doctor’s, with one participant commenting,

They should have [to] wear a white coat or something like that.

P15

The credibility of ALEX decreased as social presence was not established due to the disconnect of how unnatural the integration of his verbal and nonverbal gestures was perceived. One participant summarized perceptions of agency across multiple cues by saying,

Um, the eyes were a little narrow and just got distracted because they were so tiny. And then the language, the mouth, and his words were out of sync, too. So, if the mouth could be bigger and then in sync, it got distracting after a while, so I just decided to listen instead of looking at it. And maybe it’s just from working in a hospital, but I wanted the doctor to be in a white coat. And you know, you guys probably went one way or another, but for me, I thought it just would have felt more natural.

P17

#### Interaction and Navigability

There was a lot of discussion concerning the VC’s appearance; yet, the Black male participants expressed “ease of use” when interacting and navigating the ALEX app, leading to interaction, responsiveness, and scaffolding heuristics. The focus groups that observed this iteration of the VC provided minimal feedback on interactivity but did discuss cues that reflected the responsive nature of the VC. One participant commented,

It was a lot of content, anticipated most of my questions…Yeah, I had a positive reaction to it.

P17

Participant perceptions surrounding the navigability of the intervention were coded in terms of ease of use, and in transitions where participants engaged with the VC through a mobile phone, participants found the app to be user friendly. According to one participant,

Um, I thought it was a good app. Interesting, a good app, um, seems to be pretty user friendly.

P18

However, another stated,

I thought it was pretty good information, it’s pretty easy to use as you progress through it and use the tabs.

P19

### Iteration 3: Second Video of ALEX

**Figure 4:**
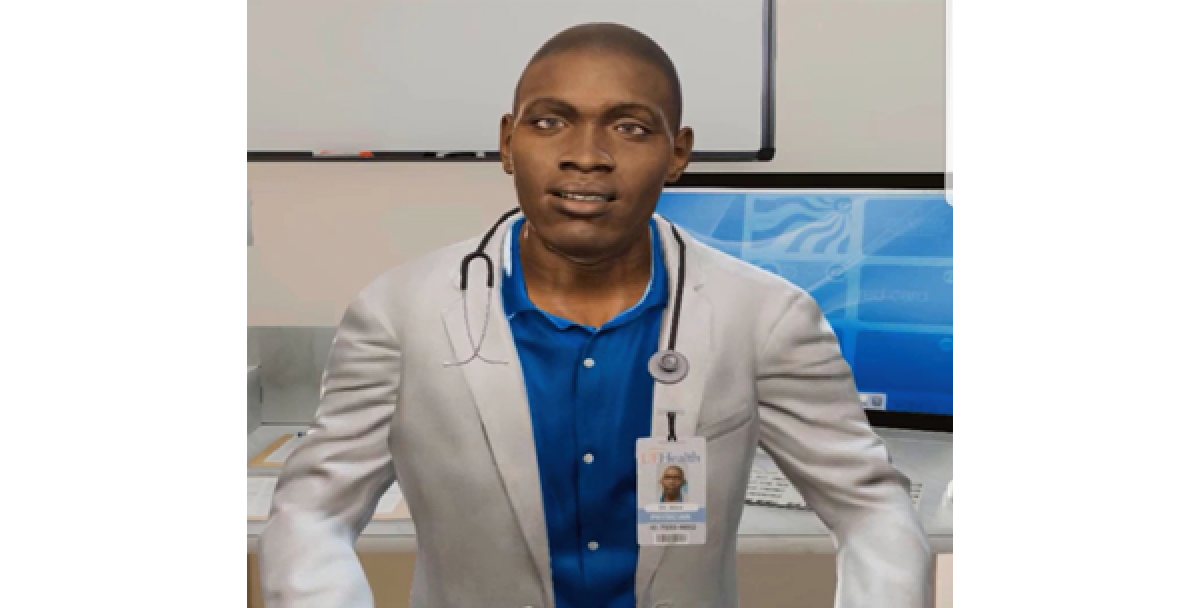
The third iteration of ALEX. ALEX: Agent Leveraging Empathy for eXams.

Feedback from the previous focus groups that examined iteration 2 of ALEX led to a re-evaluation and development of the overall agent. This redevelopment of ALEX transpired over 9 months. The VC was further modified by a 3D artist to improve the appearance and match the health care providers' clothing. Nonverbal animations were recorded by recruiting gender-matched actors using the Vicon motion capture system. The virtual character’s voice was recorded by professional voice talents who were race- and gender-matched with the virtual character. To make the environment like a real health care provider’s office, we used a background image of a real clinical room. Changes were made to the Black male VC’s eye shape and color, mouth, figure, hair, and posture, and more realistic movements were added to improve anthropomorphism. He was given props, such as a lab coat, name tag, and stethoscope, to convey an authoritative level of medical expertise, as shown in Figure 4. Facial expressions and hand gestures were added to the VC so that he could emotionally connect with the user.

#### Modality and Agency

During the third iteration, although participants were largely positive about the inclusion of multiple modalities, concerns about a lack of coordination between modalities arose that could undermine the credibility of the app. In particular, participants were distracted by the fact that the audio and visual components (ie, lip synching) were mismatched. Participants made comments illustrating this idea, such as,

I stopped watchin’ it and just started reading.

*P* 124

You can’t listen and watch his mouth.

P121

Once I looked at it, that this thing was not human, it just made me turn off, although I listen at what it was sayin’. See, I couldn’t see his body movements and the rhythm of his body…Is that thing computer generated? Because that’s not a real person. I think it oughta have a real person do it.

P119

The negative feedback about the technology and the need to improve the quality of the interaction led to the moderator raising a question concerning adding a real doctor to the app to increase credibility and acceptability. This suggestion was well received by the participants:

I think that would be great.

P119

#### Interaction and Navigability

The focus groups that observed the third irritation of ALEX provided minimal feedback on the interaction and navigability outside of not wanting to look at the VC. They expressed that navigation through the app was “simple” [P126].

### Iteration 4: Third Video of ALEX

**Figure 5:**
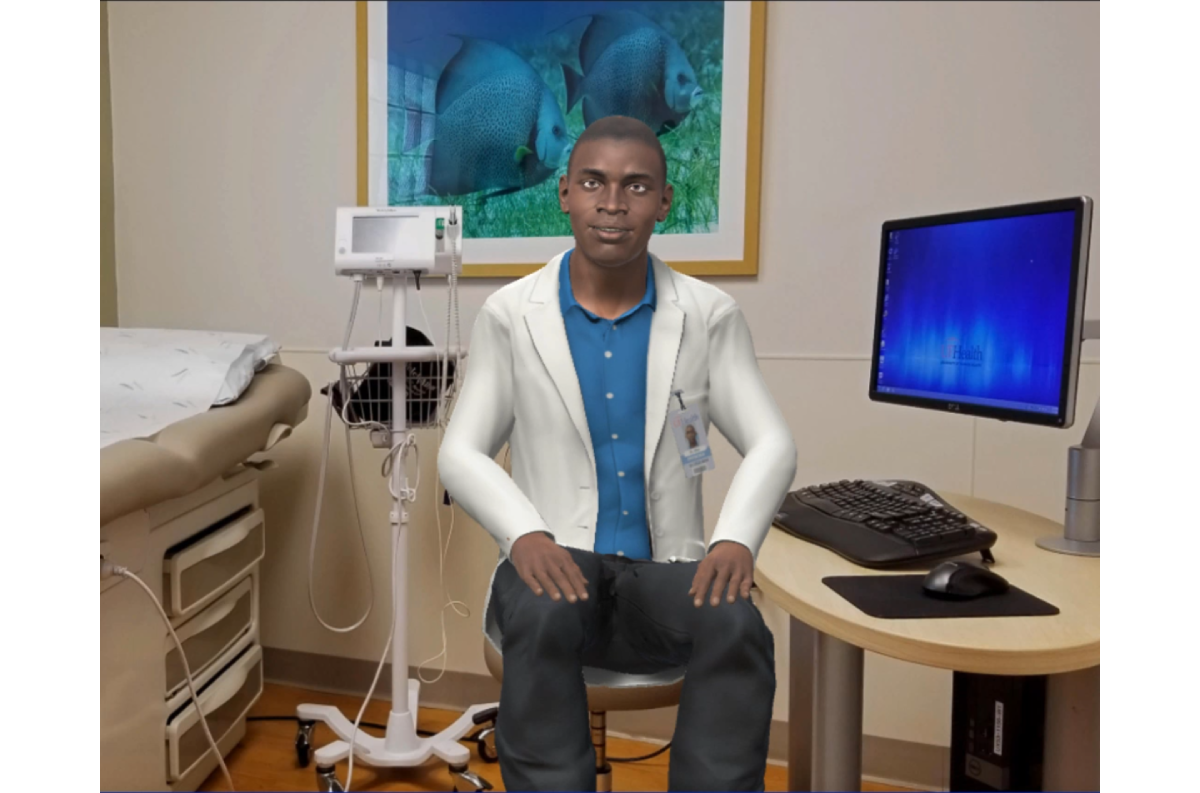
The final Iteration of ALEX. ALEX: Agent Leveraging Empathy for eXams.

The final focus group viewed the fourth iteration, which was the third and final version of the ALEX app, as shown in Figure 5, and the alterations were well received. Recommendations and comments from the previous focus groups concerning ALEX’s movement and facial expressions were incorporated to increase the humanistic behavior of the VC. As the script was read by the hired voice actors, their facial expressions were recorded and the computer science team of the project applied the movements to ALEX, focusing on eye contact and mouth movements. The spatial usage of the patient room was also enhanced with a picture on the wall, as recommended by a participant, and an upgraded computational setup, and a patient bed was added for a more realistic experience, as shown in Figure 5. Near and far recordings were aligned with different parts of the script to assist with an enhanced interaction. In addition, a video of African American male physician introducing ALEX, virtual human technology, and the purpose of the intervention was added to the app to address previous concerns and comments addressing not using a person to deliver the intervention. This enabled changes in the script that allowed enabled ALEX to focus on CRC instead of having to explain the concept of a virtual human.

#### Modality

The feedback on the multiple modalities was positive, and the participants expressed satisfaction with being able to listen, watch, and read the material. One participant stated,

I like it because some people can’t read fast enough and get it that way. They can watch a visual and soak it up better or watchin’ the video. I thought it was great.

P154

Another participant stated that they preferred listening to the VC:

By me listening and listening and—I learn a lot about colon cancer, things that I didn’t know.

P148

#### Agency

By the fourth iteration, the VC had undergone substantial revision. Revision focused on improving the VC to invoke heuristics that included trustworthiness (through appearance and voice), expertise (clothing and visual field), and social presence (movement). Social presence, through natural movement, of the VC improved when verbal and nonverbal gestures were paired together. This was further strengthened when participants finally acknowledged gender and racial matching of the VC. The men’s acceptance of ALEX reflected both outgroup (ie, a health care provider who was an authority) and in-group (social group membership through the same race and gender) characteristics, as expressed by one participant stating,

Now, me and the virtual guy, we partners…I wasn’t interested until I saw virtual man.

P152

Other participants reflected this group membership combined with greater social presence by saying,

I thought he was pretty cool, since he was a brother…I mean, he gave some informative information, especially about the FIT. It was like watchin’ a little cartoon like—Fat Albert-type situation, except he’s talkin’ more directly to a person or whatever.

P150

#### Interaction

Credibility, likeability, identity, and social presence were all reached by the fourth iteration of the VC. Feedback concerning ALEX and interacting with ALEX was expressed as “natural,” where the VC was life-like, as stated here:

I thought he was a real guy for a second.

P154

He did a lotta hand gestures, though, that’s why it’s so natural.

P150

The men described the interaction as straightforward and as a conversation where ALEX “don’t beat around the bush neither” [P149] and “Tell it like it is” [P148]. The VC was seen as an expert and authoritative figure with a medical background:

I could talk to him better than I can a live doctor.

P151

#### Navigability

There were no comments from participants regarding the navigability of the app.

### Content and Intended Behavior Outcomes from the ALEX App

One of the interesting outcomes from all focus groups was the attitude change toward screening after engaging with ALEX and learning more about CRC and the FIT kit. The content itself was novel to the men, and participants were interested in learning more, talking to their doctors, and sharing the ALEX app with others. One participant who viewed iteration 3 commented,

I’m gonna recommend that website. I’m gonna show you what I learned.

P109

It was important for the men to get the message about CRC screening out to those who could benefit:

Everybody needs that message. We’re talkin’ about it today cause it’s important. You know what I mean? I don’t want nobody to go without hearin’ the message. I would wanna share it…It’s stuff you might not get from your doctor.

P126

Specifically, the VC app was seen as supplemental information that may not be obtained otherwise.

The novelty of the app was engaging and helped men see the benefits of CRC screening:

Like I said, about the FIT, I didn’t know, okay? I’m gonna get it done. I wanna get it done…In a heartbeat.

P154

The demonstration of how to use the FIT kit was well received and informed one of the participants who had the kit at home but did not know how to use it”

In the application, when you put the thing across the seat—cause I didn’t know that. That’s why I haven’t really been botherin’ with the thing. But now, I might try and it put across the seat…If I find out, from the stool, that I need to go and get the camera up in my bottom, then that’s okay—I needed to know cause I got the test at home, but I just didn’t know how to do it.

P152

For other men, this information was viewed as novel:

I didn’t know about the FIT. Most men are embarrassed about goin’, gettin’ colonoscopies done.

P154

In addition, it provided a new way of looking at CRC screening:

This video, this app will open people eyes. It opened mine.

P148

## Discussion

### Principal Findings

In this study, Black men informed the development of a Black male VC, ALEX, that provided education on CRC, at-home screening recommendations, and risk factors. Participants influenced the visual, audio, and textual content of this app, ensuring it would be attractive and informative for future men who would interact with this app as part of a clinical trial.

The MAIN model was applied to this study to assess the credibility of ALEX. Feedback from the participants was categorized according to the four affordances.

One unique aspect of this study was the overlapping heuristic cues identified. Traditionally specific heuristic cues were associated with specific affordances [20,21,27,28]. Some heuristics such as social presence and identity were found to be associated or cued by both agency and interaction affordances in different mediums [20,21,27,28]. In this study, multiple cues triggered overlapping heuristics that led to overall enhanced user perception and credibility judgement with the VC and usage of the FIT kit. The converging cues triggered multiple heuristics associated with multiple affordances. The heuristics then led to positive qualities, including expertise, understandability, believability, clarity, importance, relevance, appearance, and representative, which were all referred to by the focus group participants at one time or another throughout this study. For instance, the multiple modalities associated with the modality affordance triggered the being there, realism, and novelty heuristics and also triggered social presence and identity when the men called ALEX their “brother-man” or would rather interact with ALEX instead of their doctor. They perceived him as a member of their inner circle because multiple modalities were provided. They could visually see ALEX and watch his movements, eye contact, and mannerisms. They could also hear the audio and his words, diction, and tone. This increased the credibility of the ALEX app.

Through their interaction with the app, the anthropomorphism of ALEX improved the most throughout the focus groups. The realism and authority heuristics were cued from the final corrections needed to establish anthropomorphism. ALEX’s movement was one of the largest distractions for the focus group members. Once the movement of the VC perfectly aligned with the audio, the men were able to focus more on the content of the app, as shown in the results.

Since the goal of this app is to encourage the user to make a positive decision regarding their health and screening for CRC, a novel affordance emerged, the content itself. The content examined the characteristics associated with conveying the right message. The men spoke of accuracy of the content related to having gone through FIT previously. The importance and understandability of the content motivated the men, and they wanted to share the app or obtain more information from their doctors. All possible cues (understandability, clarity, accuracy) for the content are examples of converging cues, where they lead to authority, helper, own-ness, and novelty heuristics associated with other affordances.

ALEX was seen both as an authority or a helper by the men, where it could serve as a resource at the doctor’s office. Others saw it as an intelligent entity and would rather engage with the app instead of a doctor. The own-ness heuristic, defined as the ability to adapt the content to the perceived interest of the users, enabled the men to make recommendations for the content and identify things that were confusing to them. We were then able to address those concerns and personalize and tailor the information for the intended users as part of the clinical trial.

The FIT kit as an option for CRC screening was highly novel to most of the focus group participants. Multiple studies have explored the methods of promoting screening via at-home fecal testing within underserved populations [6,7,33-36]. A systematic review published in 2018 identified 27 unique CRC-screening interventions using FIT or the fecal occult blood test with low-income and rural populations in different settings [36]. The authors identified the most effective studies focused on increasing community access to fecal testing, increasing or providing delivery of testing kits, developing tailored decision aids promoting screening, or interacting with patient navigators, clinicians, or community members to increase understanding of fecal testing [36]. The ALEX app captures all of those efforts in one setting.

ALEX serves as a virtual patient navigator that was designed by the targeted population, promotes screening, and provides delivery of the testing kit. Overall, the novelty and coolness of the ALEX app promoting FIT kits was well received and led to increased interest in screening, which is the goal of the app. The Black men in our study were not only interested in the FIT kit after interacting with ALEX but also wanted to share the app. This interface was liked and found trustworthy by the Black male focus group participants.

### Conclusion

In this study, the feedback from Black men helped enhance the design of the ALEX app. Future implications of the ALEX app include involvement as part of a clinical trial to deliver tailored messages promoting FIT uptake. This study entailed a great deal of reflexivity on the part of the research team. The current social climate has made direct conversations around race challenging, which is one reason we think this work is so important. The research team is racially and ethnically diverse, and we engaged in weekly conversations about both the conduct of the research study as well as the larger context. We also drew on the experience and wisdom of a racially and ethnically diverse community advisory board. We have built this relationship over the past 4 years, which enabled us to have direct and meaningful conversations about the methods as well as the results.

As digital health care interventions continue to advance, involving specific populations in the design and development of these technologies is critical. Targeted interventions, such as the one in this study, can lay the groundwork for engaging Black men in science. Long term, it can increase participation in health care interventions by promoting trust, inclusion, and responsiveness.

## Abbreviations

ALEX: Agent Leveraging Empathy for eXams
CRC: colorectal cancer
FIT: fecal immunochemical test
GPS: Global Positioning System
IRR: interrater reliability
MAIN: modality, agency, interactivity, and navigability
UCD: user-centered design
VC: virtual clinician
